# Atypical anti-glomerular basement membrane disease: two case reports

**DOI:** 10.1093/omcr/omaf073

**Published:** 2025-06-27

**Authors:** Zaineb Kaouiri, Nada El Kadiri, Loubna Benamar, Tarik Bouattar, Lamyaa Rouas, Naima Ouzeddoun

**Affiliations:** Department of Nephrology-Dialysis-Kidney transplantation, Ibn Sina Hospital, Lamfadel Cherkaoui Street, Agdal District, Rabat 10100, Morocco; Faculty of medicine and pharmacy, Mohammed V University, Avenue Mohammed Belarbi El Alaoui, Rabat 10100, Morocco; Department of Nephrology-Dialysis-Kidney transplantation, Ibn Sina Hospital, Lamfadel Cherkaoui Street, Agdal District, Rabat 10100, Morocco; Faculty of medicine and pharmacy, Mohammed V University, Avenue Mohammed Belarbi El Alaoui, Rabat 10100, Morocco; Department of Nephrology-Dialysis-Kidney transplantation, Ibn Sina Hospital, Lamfadel Cherkaoui Street, Agdal District, Rabat 10100, Morocco; Faculty of medicine and pharmacy, Mohammed V University, Avenue Mohammed Belarbi El Alaoui, Rabat 10100, Morocco; Department of Nephrology-Dialysis-Kidney transplantation, Ibn Sina Hospital, Lamfadel Cherkaoui Street, Agdal District, Rabat 10100, Morocco; Faculty of medicine and pharmacy, Mohammed V University, Avenue Mohammed Belarbi El Alaoui, Rabat 10100, Morocco; Faculty of medicine and pharmacy, Mohammed V University, Avenue Mohammed Belarbi El Alaoui, Rabat 10100, Morocco; Department of anatomopathology, Ibn Sina Hospital, Lamfadel Cherkaoui Street, Agdal District, Rabat 10100, Morocco; Department of Nephrology-Dialysis-Kidney transplantation, Ibn Sina Hospital, Lamfadel Cherkaoui Street, Agdal District, Rabat 10100, Morocco; Faculty of medicine and pharmacy, Mohammed V University, Avenue Mohammed Belarbi El Alaoui, Rabat 10100, Morocco

**Keywords:** atypical anti-glomerular basement membrane disease, crescentic glomerulonephritis, Goodpasture, IgG linear deposits

## Abstract

Atypical Anti-Glomerular Basement Membrane (anti-GBM) Disease, a variant of the rare autoimmune disorder Goodpasture’s disease, presents unique challenges in diagnosis and management. This article presents two cases of atypical anti-GBM disease characterized by negative serological tests for anti-GBM antibodies, minimal renal impact on kidney biopsy, and absence of pulmonary involvement. These cases underscore the evolving spectrum of renal anti-GBM-related conditions and the need for a nuanced approach to diagnosis and treatment. Treatment with high-dose steroids resulted in favorable outcomes in both cases, highlighting the importance of individualized management strategies, especially given the absence of standardized recommendations for this uncommon presentation. Further research is warranted to elucidate the pathophysiology and optimal management of atypical anti-GBM disease.

## Introduction

Anti-glomerular basement membrane (anti-GBM) disease, also referred to as Goodpasture’s disease, is a rare autoimmune disorder affecting both the kidneys and lungs. It involves the production of antibodies targeting the glomerular basement membrane (GBM) and the alveolar basement membrane in the lungs [1]. These antibodies, targeting the alpha-3 chain of type IV collagen, induce inflammation and tissue damage. The disease most commonly presents with kidney involvement (crescentic glomerulonephritis) and lung involvement (pulmonary hemorrhage) [2].

The exact cause of anti-GBM disease is not fully understood but is believed to result from a combination of genetic predisposition and environmental triggers. Diagnosis typically involves blood tests to detect anti-GBM antibodies, kidney biopsy to identify linear IgG deposition along the GBM, and imaging studies to assess organ involvement. Treatment generally includes immunosuppressive medications, such as corticosteroids and cyclophosphamide, to suppress the immune response and reduce inflammation. Plasma exchange is often employed to remove circulating antibodies. Prompt and aggressive treatment is essential to prevent irreversible damage to the kidneys and lungs [3].

The prognosis varies widely, with early detection and treatment significantly improving outcomes. However, in some cases, the disease can still be life-threatening. Recently, cases have been reported featuring linear IgG deposition along the GBM with distinct clinical and pathological features. These cases, which may differ in prognosis and presentation, have been referred to as ‘atypical anti-GBM disease,’ expanding the spectrum of renal anti-GBM-related conditions.

## Case report

### Case one

A 46-year-old man with no significant medical history, a chronic active smoker, experienced an episode of tonsillitis one week prior to symptom onset. He presented to nephrology with an edematous syndrome.

On physical examination, the only notable finding was edema, with normal blood pressure. Laboratory tests revealed a nephrotic syndrome (albuminemia: 22 g/l, proteinuria: 4.9 g/24 h), elevated creatinine (2.8 mg/dl), and gross hematuria. Screening for autoantibodies (anti-nuclear, anti-DNA, anti-neutrophilic cytoplasmic, and anti-GBM by ELISA) was negative, with normal levels of IgA, IgG, and IgM antibodies. Complement levels (C3, C4) were within normal ranges (Table 1). Additional laboratory tests, including glycemia, hemoglobin A1c, and serological tests for viral hepatitis and HIV, were also negative.

**Figure 1:**
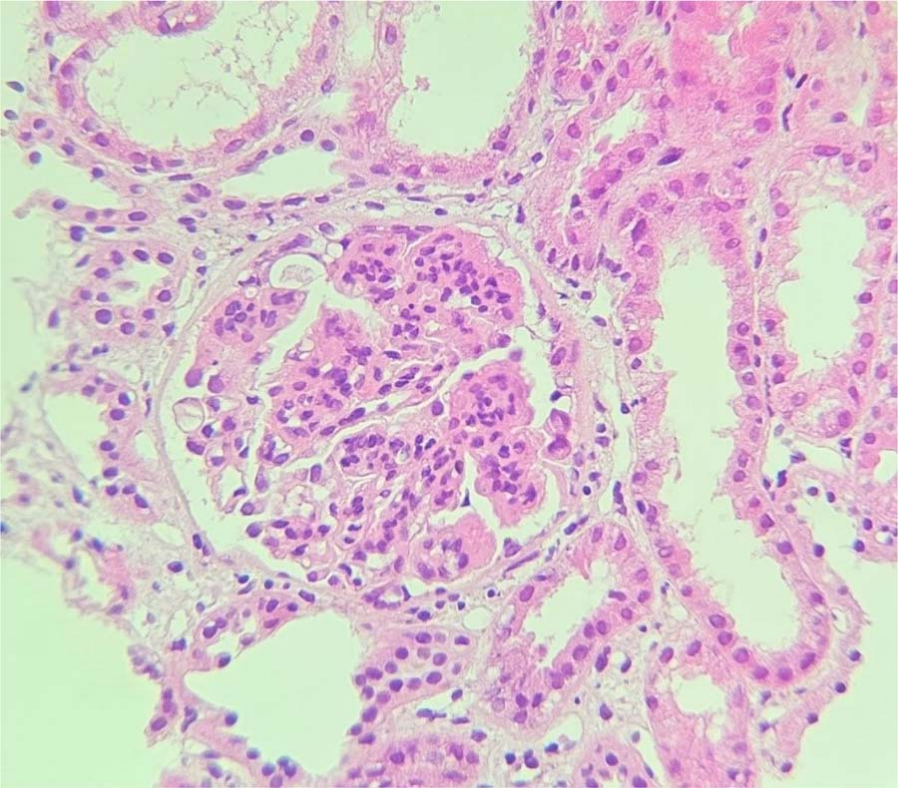
Glomerulus. H&E stain (x40) showing global mesangial expansion. (case 1).

**Table 1:** Laboratory results (chemistry, urinalysis, complete blood count, immunological tests) on admission and at 6 months follow up. (case 1).

	Case one		Reference Range
	Admission	6 months	
AST (U/l)	13	16	5–34
ALT (U/l)	7	10	0–55
Total protein (g/l)	50	65	64–83
Serum Albumin (g/l)	22	34	35–52
Blood Urea (g/l)	0.34	0.22	0.15–0.55
Creatinine (mg/l)	21.1	10	5.7–12.5
eGFR (ml/min/1.73 m2)	34	85	>60
Na^+^ (mEq/l)	138	141	136–145
K^+^ (mEq/l)	4.3	4.2	3.5–5.1
Cl^−^ (mEq/l)	107	103	98–107
Calcium (mg/l)	96	93	84–102
Phosphate (mg/l)	43	45	23–47
Uric Acid (mg/l)	39	42	26–60
Plasma glucose (g/l)	0.9	0.83	0.7–1.1
TSH us (μUI/ml)	1.96	-	0.35–4.94
Lactate dehydrogenase (U/l)	186	-	0–250
Ferritin (ng/mL)	10	-	4–204
Hemoglobin A1c (%)	5	-	<5.7%
Urine red blood cell (/mm3)	78	4	<15
Urine White cells (/mm3)	20	0	<10
Proteinuria (g/d)	4.9	0.28	<0.3
White blood cell (/μl)	10 100	9700	4000–10 000
Neutrophil (%)	5062	6397	1500–7000
Lymphocyte (%)	3939	2910	1000–4000
Eosinophil (%)	181	106	100–400
Basophil (%)	141	87	0–100
Monocyte (%)	777	200	200–1000
Hemoglobin (g/dl)	12.1	12.8	13–16.5
Platelet (/μl)	328 000	208 000	150 000–400 000
IgA dosage (g/l)	1.37	-	0.63–4.84
IgG dosage (g/l)	8.29	-	5.4–12.22
IgM dosage (g/l)	1.82	-	0.22–2.4
C3 (g/l)	1.13	-	0.83–1.93
C4 (g/l)	0.24	-	0.15–0.57

Abdominal ultrasonography revealed normally sized, well-differentiated kidneys, while thoracic CT scan revealed minimal bilateral pleurisy consistent with nephrotic syndrome.

**Figure 2:**
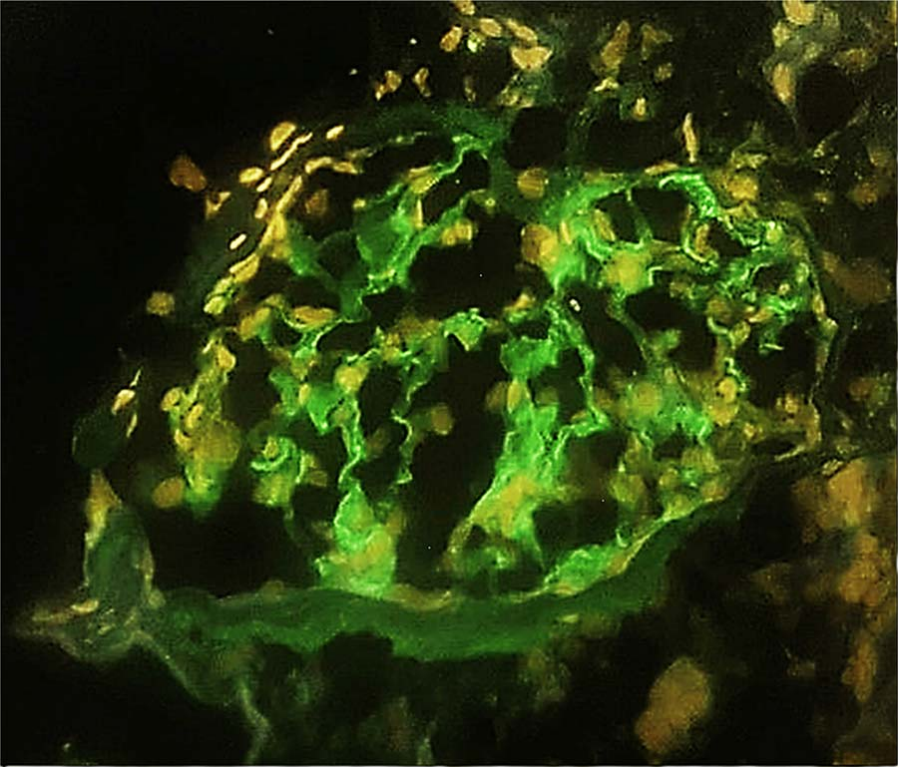
IgG immunofluorescence staining shows linear staining of GBMs. (case 1).

Kidney biopsy findings under light microscopy included 10 glomeruli with no global sclerosis, no crescents, segmental endocapillary and mesangial hypercellularity, and one glomerulus with a fibrin thrombus. There was moderate focal interstitial inflammation, but no interstitial fibrosis or tubular atrophy (Figure 1). Immunofluorescence revealed diffuse, linear, polytypic IgG deposition along the GBM and pseudolinear C3 staining (2+) (Figure 2).

Based on the absence of clinical or radiological lung involvement, negative anti-GBM serology, and minimal renal involvement, the diagnosis of atypical anti-GBM disease was made. The patient was treated with high-dose steroids (15 mg/kg/day for 3 days), which resulted in decreased proteinuria and hematuria and improved renal function.

### Case two

A 32-year-old man, also a chronic active smoker, experienced tonsillitis two weeks prior to symptom onset. He presented to nephrology with gross hematuria.

Physical examination was unremarkable except for macroscopic hematuria. Laboratory tests showed elevated creatinine (8 mg/dl), proteinuria (2.65 g/day), and gross hematuria. Autoantibody screening (anti-nuclear, anti-DNA, anti-neutrophilic cytoplasmic, and anti-GBM by ELISA) was negative, with normal IgA, IgG, and IgM levels. Complement levels (C3, C4) were normal (Table 2), and additional tests, including glycemia, hemoglobin A1c, and serologies for viral hepatitis and HIV, were negative.

**Table 2:** Laboratory results (chemistry, urinalysis, complete blood count) on admission and at 6 months follow up. (case 2).

	Case 2		Reference Range
	Admission	6 months	
AST (U/l)	17	13	5–34
ALT (U/l)	9	11	0–55
Total protein (g/l)	61	67	64–83
Serum Albumin (g/l)	34	42	35–52
Blood Urea (g/l)	0.98	0.55	0.15–0.55
Creatinine (mg/l)	64	16	5.7–12.5
eGFR (ml/min/1.73 m2)	10	49	
Na^+^ (mEq/l)	135	141	136–145
K^+^ (mEq/l)	4.9	4.1	3.5–5.1
Cl^−^ (mEq/l)	99	109	98–107
Calcium (mg/l)	90	90	84–102
Phosphate (mg/l)	25	31	23–47
Uric Acid (mg/l)	62	60	26–60
Plasma glucose (g/l)	0.7	0.79	0.7–1.1
TSH us (μUI/ml)	0.88	-	0.35–4.94
Lactate dehydrogenase (U/l)	262	-	0–250
Ferritin (ng/ml)	200	-	4–204
Hemoglobin A1c (%)	5.2	-	<5.7%
Urine red blood cell (/mm3)	5	2	<15
Urine White cells (/mm3)	44	1	<10
Proteinuria (g/d)	2.65	0.15	<0.3
White blood cell (/μl)	9390	9600	4000–10 000
Neutrophil (%)	4861	4671	1500–7000
Lymphocyte (%)	3725	3900	1000–4000
Eosinophil (%)	140	362	100–400
Basophil (%)	56	95	0–100
Monocyte (%)	608	572	200–1000
Hemoglobin (g/dl)	13.2	12.8	13–16.5
Platelet (/μl)	324 000	307 000	150 000–400 000
IgA dosage (g/l)	2.09	-	0.63–4.84
IgG dosage (g/l)	7.1	-	5.4–12.22
IgM dosage (g/l)	1.5	-	0.22–2.4
C3 (g/l)	1.44	-	0.83–1.93
IgA dosage (g/l)	2.09	-	0.63–4.84

**Figure 3:**
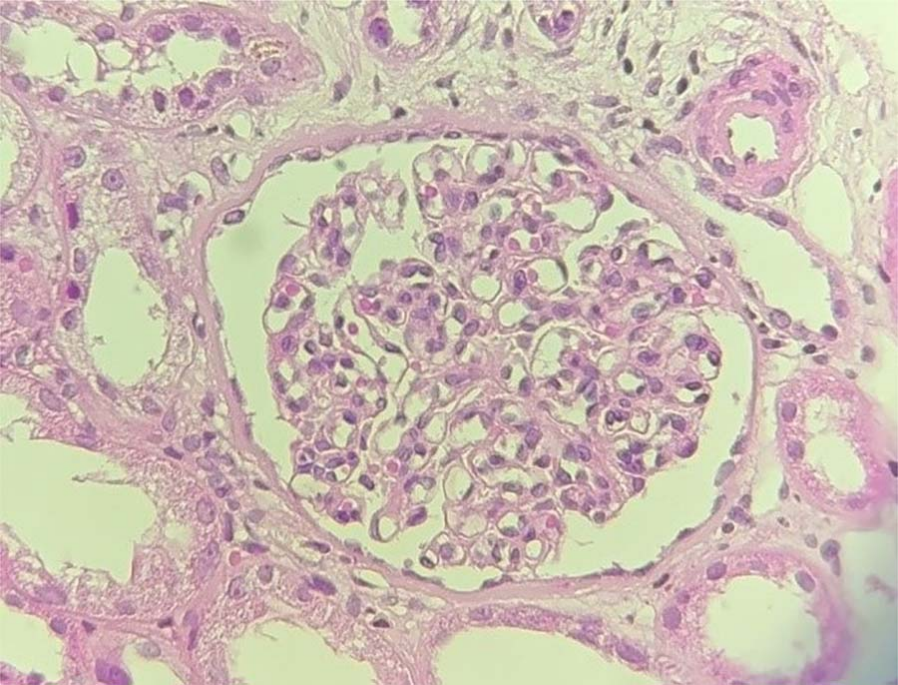
Glomerulus. H&E stain (x40) showing global mesangial expansion. (case 2).

**Figure 4:**
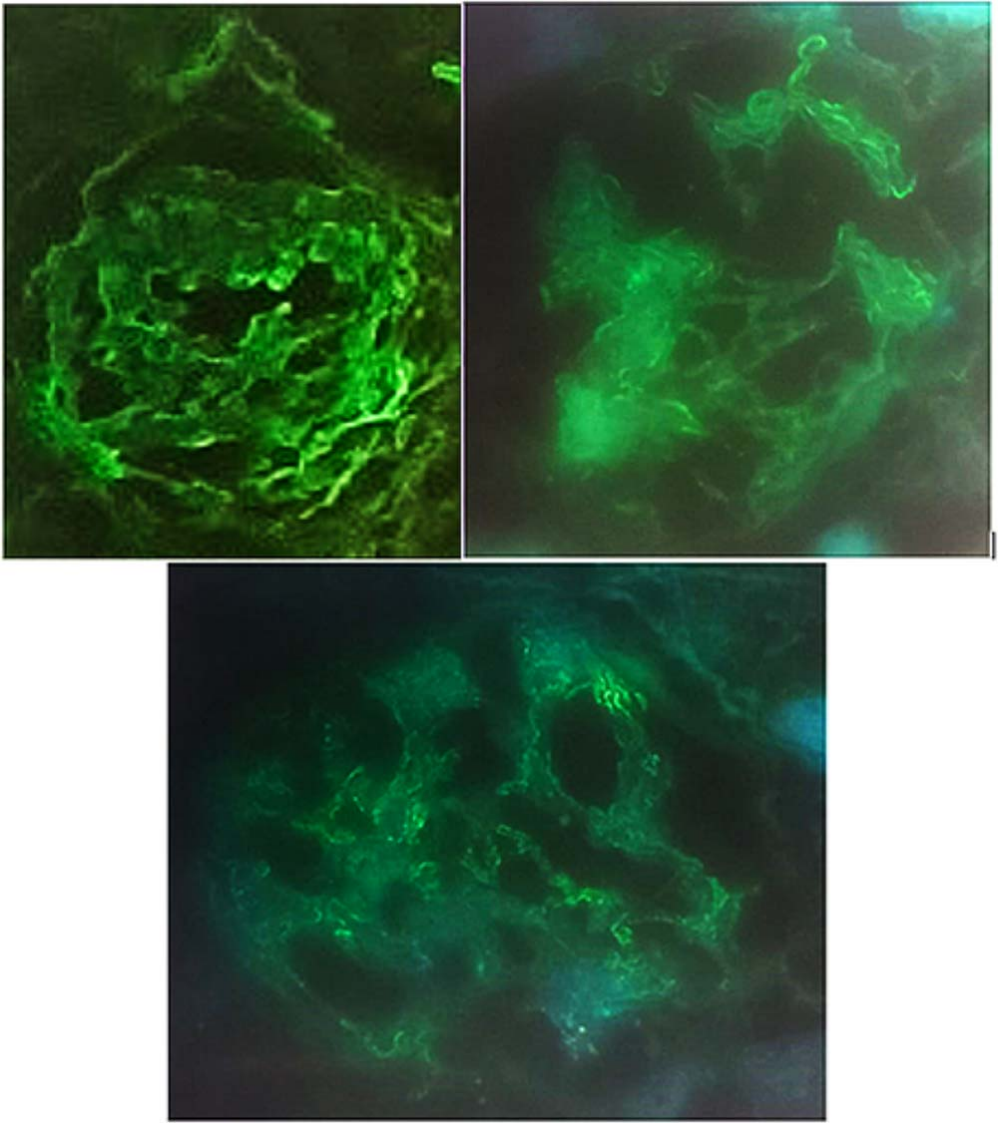
IgG immunofluorescence staining shows linear staining of GBMs. (case 2).

Abdominal ultrasonography revealed normal kidney size and differentiation, while thoracic CT scan showed no evidence of alveolar hemorrhage.

Kidney biopsy findings included segmental mesangial thickening and hypercellularity, segmental endocapillary hypercellularity, and no crescents. Moderate interstitial inflammation, as well as moderate interstitial fibrosis and tubular atrophy, were observed (Figure 3). Immunofluorescence revealed thin, linear, polytypic IgG staining along the GBM, along with IgM and C1q deposition (Figure 4).

Given the lack of pulmonary involvement, negative anti-GBM serology, and minimal renal impact, a diagnosis of atypical anti-GBM disease was made. Treatment with high-dose steroids (15 mg/kg/day for 3 days) resulted in decreased proteinuria and hematuria and improved kidney function.

In both cases, potential differential diagnoses for IgG staining along the GBM, such as diabetic nephropathy, membranous nephropathy, and monoclonal immunoglobulin deposition disease, were systematically excluded through clinical, biological, and pathological investigations.

In both cases, other potential causes of IgG staining on the glomerular basement membrane (GBM) were systematically excluded through a combination of clinical, biological, and pathological investigations. Diabetic nephropathy was ruled out based on normal glycemic values, normal hemoglobin A1c levels, and the absence of diabetic retinopathy confirmed by a comprehensive ophthalmological examination. Membranous nephropathy was excluded due to the lack of extra-membranous IgG deposits on immunofluorescence microscopy. Monoclonal immunoglobulin deposition disease was eliminated by demonstrating the absence of monoclonal heavy and/or light chains in the kidney tissue and by finding no abnormalities in serum protein electrophoresis and serum protein immunofixation studies. These thorough investigations, combined with the clinical and histopathological context, allowed us to confidently rule out these differential diagnoses.

## Discussion

Anti-GBM disease is a small vessel vasculitis, classified as an immune-complex small vessel vasculitis in the Chappel Hill classification. It is characterized by circulating antibodies targeting basement membrane antigens, thus, affecting kidneys and lungs [4]. Its frequency is of 0.5 to two cases per million per year in European and Asian populations [5]. Anti-GBM disease remains very rarely reported in African population [6]. Atypical anti-GBM disease have been reported in Wilson and Dixon’s 1973 report, by describing the case of IgG linear deposition on a kidney biopsy done during splenectomy, treated with steroids, with great response [7]. The largest study to date, reporting 60 cases of atypical anti-GBM disease, has been published by Shen CR et al. in 2020 [8]. These uncommon cases show slightly different or completely divergent features, creating a real spectrum of renal anti-GBM related conditions. They can differ from the classical form in clinical characteristics, prognosis, serological features, and histological findings.

Classic anti-GBM disease usually presents as a nephritic syndrome or rapidly progressive glomerulonephritis (80%–90%), with pulmonary hemorrhage (40%–60%), or without it [1]. Only few patients may have isolated pulmonary lesions. Our first patient presented with nephrotic syndrome, which is very rarely seen in classical anti-GBM disease, associated with hematuria, and a slowly increasing creatinine unlike the rapidly progressive renal failure observed in other cases. The second patient also presented with a slowly increasing creatinine associated with hematuria.

Furthermore, many patients may report an infectious episode (flu, tonsillitis…for example), before the actual disease, like our two patients’ histories of tonsillitis that preceded the onset of the renal symptoms. In addition to the clinical features cited below, the diagnosis of anti-GBM disease is based on the identification of anti-GBM antibodies in serum or deposited along the GBM in kidney biopsy. The serological test for anti-GBM antibodies were negative in our cases, but the kidney biopsy showed thin linear IgG staining along GBMs without crescents. These features not only confirm the anti-GBM entity and its atypical nature, but also indicate a potentially favorable renal and vital prognosis (absence of active lesions as crescentic glomerulonephritis).

Several hypotheses have been proposed to explain the phenomenon of linear Ig deposition observed in atypical cases of anti-GBM. One theory suggests the presence of autoantibodies against GBM antigens, which may not be detectable through standard serological tests, resulting in false negative results. Another hypothesis posits a potential physicochemical attraction between GBM collagen and certain monotypic light chains. Lastly, a third hypothesis revolves around the physicochemical alterations of collagen molecules, particularly the loss of negative charges, leading to the deposition of circulating proteins, predominantly IgG4 and albumin [9].

In classical anti-GBM cases, a combination of high-dose steroids, cyclophosphamide, and plasmapheresis is employed [10]. However, the risk–benefit ratio of these aggressive regimens remains questionable for patients with atypical anti-GBM disease, who often exhibit less severe clinical and histological manifestations and may lack pulmonary hemorrhage. Within the Shen CR and al. cohort, the diverse clinical and histological features of patients with atypical anti-GBM disease resulted in different treatment regimens. This diversity made it impossible to prove the correlation between immunosuppressive therapy and renal outcomes in the study [8].

In the presented cases, the patients exhibited no pulmonary hemorrhage, and renal function was gradually declining. Treatment with high-dose steroids successfully decreased proteinuria, hematuria, and improved renal function. Currently, there are no standardized recommendations for the treatment of atypical anti-GBM disease due to the considerable heterogeneity among these patients. Treatment approaches vary widely and heavily rely on clinical judgments made by physicians. After a six months follow-up period, both patients exhibited negative proteinuria and hematuria, along with improved kidney function. Given the significant heterogeneity among atypical anti-GBM patients, follow-up protocols and results, varied widely among individuals based on treatment regimens and their clinical and histological characteristics.

## Conclusion

Anti-glomerular basement membrane disease, though rare, presents a critical challenge due to its potential to severely impact renal and pulmonary systems. The emergence of atypical anti-GBM disease, as evidenced by the two cases described, highlinghts the necessity for heightened clinical awareness and nuanced diagnostic approaches.

Treatment with high-dose steroids proved effective in managing proteinuria and hematuria, improving renal function in both patients. However, the lack of standardized treatment protocols for atypical anti-GBM disease necessitates individualized therapeutic strategies.
